# Attitudes towards transactional data donation and linkage in a longitudinal population study: evidence from the Avon Longitudinal Study of Parents and Children

**DOI:** 10.12688/wellcomeopenres.15557.2

**Published:** 2021-06-09

**Authors:** Anya Skatova, Kate Shiells, Andy Boyd

**Affiliations:** 1 School of Psychological Science, University of Bristol, Bristol, BS8 1TU, UK; 2 The Alan Turing Institute, London, NW1 2TB, UK; 3 Bristol Medical School, Population Health Sciences, University of Bristol, Bristol, BS8 2BN, UK

**Keywords:** ALSPAC, attitudes, banking data, retail records, consent, data linkage, longitudinal research, safeguards

## Abstract

**Background:** Commercial transaction records, such as data collected through banking and retail loyalty cards, present a novel opportunity for longitudinal population studies to capture data on participants’ real-world behaviours and interactions. However, little is known about participant attitudes towards donating transactional records for this purpose. This study aimed to: (i) explore the attitudes of longitudinal population study participants towards sharing their transactional records for health research and data linkage; and (ii) explore the safeguards that researchers should consider implementing when looking to request transactional data from participants for data linkage studies.

**Methods:** Participants in the Avon Longitudinal Study of Parents and Children were invited to a series of three focus groups with semi-structured discussions designed to elicit opinions. Through asking participants to attend three focus groups we aimed to facilitate more in-depth discussions around the potentially complex topic of data donation and linkage. Thematic analysis was used to sort data into overarching themes addressing the research questions.

**Results:** Participants (n= 20) expressed a variety of attitudes towards data linkage, which were associated with safeguards to address concerns. This data was sorted into three themes: understanding, trust, and control. We discuss the importance of explaining the purpose of data linkage, consent options, who the data is linked with and sensitivities associated with different parts of transactional data. We describe options for providing further information and controls that participants consider should be available when studies request access to transactional records.

**Conclusions:** This study provides initial evidence on the attitudes and concerns of participants of a longitudinal cohort study towards transactional record linkage. The findings suggest a number of safeguards which researchers should consider when looking to recruit participants for similar studies, such as the importance of ensuring participants have access to appropriate information, control over their data, and trust in the organisation.

## Introduction

Data linkage, or the linking of two or more different sources of information about the same phenomena of interest ^1^, is an efficient and cost-effective method for carrying out epidemiological research ^2^. It is particularly beneficial for longitudinal cohort studies, an observational research method where data about the same participants are gathered repeatedly over a period of years or even decades ^3^, as it allows for the study of links between a vast range of behaviours, medical conditions, environmental factors, genes, lifestyle choices and health outcomes ^4^. Whilst cohort studies use advanced data collection protocols, a large amount of information on the daily behaviours and lifestyles of participants is collected by self-report and hence subject to missingness and/or bias ^5^. Objectively recorded routine records provide a means to quantify and address these concerns. For this reason, funders of UK longitudinal population studies (LPS) have identified record linkage as a strategic priority, and within this are encouraging studies to investigate linkages to a wide range of novel sources ^6– 8^.

Commercially collected transactional records, such as banking, phone, internet and retail loyalty card records, present a novel opportunity for LPS to collect objective information on participants’ behaviours. However, contemporary data science approaches recognise that using potentially sensitive information such as these needs to be based on rigorous co-design frameworks involving a wide range of stakeholders: insights from this process can then be used to identify the bounds and safeguards needed to make the data use acceptable, and to use diplomacy traditions and means to help reconcile stakeholder views into an acceptable data use framework ^9– 11^.

As a first step towards developing an ethical and privacy preserving framework for linkage of commercial transactional datasets into LPS databanks, this paper uses focus groups to investigate the attitudes and understanding of participants in the Avon Longitudinal Study of Parents and Children (ALSPAC).

### Public attitudes towards the donation of personal data

One of the principal aims of the General Data Protection Regulation ^12^ is to afford greater control over personal data to the individual ^13^, and the right to data portability (Article 20) has provided a legally mandated mechanism that can be used for the general public to obtain and donate their individual digital footprint data (an individual’s trail of online data ^14^) for research purposes ^15^. We refer to data donation as ‘an act of active consent of an individual to donate their personal data for research’ ^16^. However, previous research has demonstrated that participants do not always have sufficient knowledge or understanding about their personal data or what they can be used for ^17, 18^, suggesting that not all individuals are well-informed to make decisions about donating personal data.

In light of these findings, there is a growing body of literature seeking to explore public attitudes towards donating personal data for research. This has mostly been in the medical records domain, although researchers have also recently begun to explore views on the use of itemised phone call records ^19^. In their systematic reviews, Aitken *et al.* ^17^ and Stockdale *et al.* ^18^ found that, despite a common lack of knowledge and awareness about the value of patient data for research, there is a general willingness to share medical data. This was linked with a sense of obligation, altruism and expectation that the data used for research will contribute towards knowledge for the greater public good. Jones *et al.* (2019) ^19^ found that only 3% of participants in their study were aware that mobile phone data was being used in health research. However, 62% supported the use of their data for this purpose. Similarly, Skatova & Goulding ^16^ demonstrated that individuals are willing to donate their loyalty card data to research benefiting the public good. The decision to donate personal data was associated with three distinct reasons: being a good member of society, prosocial motivation and understanding the reasons for donating personal data.

However, others ^20– 23^ have demonstrated that there is a sense of fear towards data donation amongst the general public, connected with concerns around hacking, identify theft and the misuse of patient data for financial gain, as well as the need to both protect individual privacy and to trust the entity with which the data is shared. Whether individuals support sharing their medical records is also dependent on factors such as confidentiality, presence of safeguards to prevent the misuse of data, perceived control over how data is used, and the opportunity to provide explicit consent for sharing data.

### Public attitudes towards data linkage

There is considerable literature around consent for data linkage for research purposes ^18, 24^. From a researcher’s perspective, obtaining consent for linkage of records is likely to require participant identification, which can be viewed as a breach of confidentiality, and non-response to consent requests could introduce sample bias ^24^. Furthermore, a longstanding counter-argument against the need for consent is that the risk of non-response could hinder scientific progress and ‘undermine the public good’ ^25^. These arguments have often been used to reject the typical opt-in consent process normally used by researchers when linking previously collected data into longitudinal population studies ^2^, in favour of opt-out consent.

The opinions of the general public on consent for data linkage appear more divided. A previous qualitative interview study exploring the views of ALSPAC participants (n=55) on consent ^26^, revealed that the type of data proposed for data linkage was an important consideration, with fears about the sensitivity of data, and whether the individual could be stigmatised. Stigmatisation could happen, for example, through the linkage of teenage pregnancy and state benefits data, and the linkage of mental health records and criminal records. For these reasons, opt-in consent was preferable amongst some participants ^26^. Similar views were found by Davidson *et al.* ^21^ in a qualitative study with workshops exploring public attitudes (n=73) towards the acceptability of cross-sectoral data linkage, such as health, social care and education data, where for instance, participants feared discriminatory treatment by agencies for having a criminal record.

The degree to which the topic or outcome of the research was considered beneficial for the public good was also influential on participant views in both studies ^21, 26^. For example, the linkage of birth weight and future health outcomes was considered by some to not require opt-in consent due to the potential benefits ^26^. Participants (n=26) in a qualitative interview study by Xafis ^27^ were also more likely to express that consent was not required when they were aware of the public benefits of proposed research projects. Furthermore, participants in this study stated that consent for data linkage was not required when they held trust in the data linkage organisation, whereas the predominant view was that consent should be sought when researchers carry out the data linkage process and have access to identifying information.

Other concerns included whether linked data could be sold for commercial or political purposes, and the increased likelihood of hacking and data misuse due to the way in which more people would have access to the data ^21^. However, fears were linked with a lack of awareness around data de-identification, and when participants were assured that identifying information would not be revealed, many were less nervous about data linkage ^21^. Likewise, confirmation about the anonymity of data influenced participant decisions about consent in the study by Audrey *et al.* ^26^, with some believing it was not necessary if data was analysed at population level.

### Aims of the study

Despite the growing research interest in public opinion on linkage of health-related data, public attitudes towards sharing transactional records for data linkage remain an unexplored area, specifically in the context of longitudinal population studies and health research. As a first step in co-designing a conceptual framework for longitudinal population studies, we invited ALSPAC participants to focus groups to collect their opinions on linking transactional records for research, specifically retail loyalty cards and banking cards data. These two data types were emphasised as ALSPAC are considering mechanisms for using these data to enable new research possibilities. We studied participant attitudes towards ALSPAC requesting access to transactional records to link with their individual data in the ALSPAC databank for use in future research. This paper presents the results of these conversations in response to the following overarching research questions:

1. What are the attitudes and concerns of ALSPAC participants towards providing consent for accessing their personal retail and banking records for linkage of these data into the ALSPAC databank?
2. What are the safeguards that should be put in place by researchers to address any concerns raised by participants?

## Methods

### ALSPAC

ALSPAC is a multigenerational prospective birth cohort study. ALSPAC recruited pregnant women resident in and around the City of Bristol (South-West UK) and due to deliver between 1st April 1991 and 31st December 1992. There were an initial 14,541 enrolled pregnancies comprising 14,676 foetuses (for these at least one questionnaire has been returned or a “Children in Focus” clinic had been attended by 19/07/99). These pregnancies resulted in 14,062 live births and 13,988 children alive at 1 year. From age 7, attempts were made to recruit additional cases who were eligible under the original sample definition ^28, 29^). By age 24, an additional 913 index children had enrolled. The total sample size for analyses using any data collected after the age of seven is therefore 15,454 pregnancies, resulting in 15,589 foetuses. Of these, 14,901 were alive at 1 year of age ^30^. The cohort has been followed intensively from birth through self-completed questionnaires and attending clinical assessment visits. ALSPAC has built a rich resource of phenotypic and genetic information relating to multiple genetic, epigenetic, biological, psychological, social, and other environmental exposures and outcomes. ALSPAC is a globally accessible research resource, which also allows recall studies: including those considering participant understanding, expectations and acceptability of different research designs. The ALSPAC resource has an online data dictionary ( http://bristol.ac.uk/alspac/researchers/our-data/) and a public access mechanism ( http://bristol.ac.uk/alspac/researchers/access/)."

### Study design

We used focus groups as a method of data collection, incorporating semi-structured discussions to elicit participant attitudes towards using transactional data for public health research, and the linking of transactional records into longitudinal population studies. The focus groups were run in three parts, each a month apart. This time gap was created to help participants digest information about personal data as well as any issues that arose in the discussion. We expected that with this time gap, participants would come to informed and deliberate opinions as to whether it is appropriate to share specific types of their personal data with ALSPAC for academic research, and also comment on more or less appropriate routes for research. Each part of the focus group had a different discussion topic. Each participant was invited to all three parts of focus groups, albeit not all participants took part in each part because of individual reasons unrelated to the study. Written materials for each part were not provided to participants in advance. 

For Focus Group Part 1 and Focus Group Part 3, two separate focus groups were conducted. For Focus Group Part 2, only one focus group was held, due to difficulties in recruiting a sufficient number of participants. Each focus group lasted between 60 and 120 minutes. Authors AS and AB conducted the focus groups with a member of the ALSPAC family participation team also present. AS is a research fellow external to ALSPAC. AB is the ALSPAC data manager but had not previously met any of the participants in this study. Focus groups were audio-recorded using dictaphones and AS took field notes as well as photographs from focus group one showing how participants had sorted various categories of data.

In Focus Group Part 1, we explored dimensions that underpin barriers for data linkage (e.g., trust in how the data will be handled, understanding what will happen with the data etc), as well as attitudes to sharing and using personal data in general, both for research and commercially. This focus group introduced participants to different types of personal data as well as issues around sharing personal data in general. Since previous research showed that individuals know little about their personal data ^17, 18^, participants were briefed on the most common types of data that can be collected about them through digital means and asked to group or order according to their own choice of categories. For instance, from least sensitive to most sensitive. Types of data presented to participants on cards included: mobile phone; car GPS; electricity use; physical activity (exercise); browsing history; search history; click history; car speed records; cycling camera video; sleep patterns; bank transactions; online shopping history; age, gender, marital status; medical records; online dating history; social media; loyalty card data; mobile phone use; broadband use; and home address. This was followed by a more specific discussion about sharing financial and retail loyalty cards records with ALSPAC for academic research. Participants alternated between working in pairs and group discussion to facilitate interaction.

In Focus Group Part 2, we explored attitudes to sharing personal data that were discussed by participants in more detail using an interactive game approach. Participants were firstly presented with a set of ‘info cards’, which explained the different elements of the record linkage process, such as ‘data protection’, ‘informed consent’, and ‘data reuse’. They were also given a set of ‘issue cards’ with various beliefs associated with record linkage such as ‘data anonymisation is a myth’ and ‘individuals want to be aware of how their data is being used’. For both sets, participants were asked to pick one or two cards, which they found interesting or controversial and explain to the group. They were then provided with ‘story cards’ which presented the viewpoints of various fictional individuals involved in or potentially affected by record linkage. For instance, an ALSPAC staff member, a policy maker and a person with a rare disease. Participants were asked to discuss whether they identified with a particular story card, or whether they found a story card controversial and present this to the group. Finally, we asked participants to make decisions on whether to grant permission to specific (hypothetical) research projects that would use data linkage and various forms of consent.

In Focus Group Part 3, we presented participants with a conceptual framework of how the linkage can be done within ALSPAC. The framework included different options that were discussed with participants: e.g. the data can be shared anonymously with authorised third-party (non-ALSPAC) academic researchers; the past retail or banking records can be linked but not the future data. We focused on the acceptability of linkage scenarios and the different ways in which the data can be shared. In particular we were interested in exploring the attitudes towards retrospective versus prospective data collection given ethical views suggesting that asking consent for retrospective data is more acceptable than asking consent for ongoing prospective harvesting of information. Full protocol of the focus groups is available on request from the first author. The focus groups were conducted in April – June 2018.

### Ethical approval

Ethical approval for the study was obtained from the ALSPAC Ethics and Law Committee and the Local Research Ethics Committees. Informed consent for the use of data collected in this study was obtained from participants following the recommendations of the ALSPAC Ethics and Law Committee at the time (a University of Bristol faculty ethics committee).

### Participants, recruitment strategy and compensation

We used convenience sampling for recruitment: a sub-set of ALSPAC participants (n=600) from the index generation were invited to participate when aged 25–26 years old. The sub-set was randomly selected from a pool of individuals with a Bristol postcode (in order to facilitate attendance at multiple workshops) and who had a valid email address on file. Standard filters were also applied, with people who had died, withdrawn, or asked for a break from participation not included in the pool.

We sent an email invitation letter and an information sheet about the nature of the study. Any invited ALSPAC index participant could take part, whether they used services like banks, held loyalty cards, or not. All contact with participants was managed by ALSPAC administrative staff, who recruited the participants and arranged suitable dates for the focus groups. Although focus groups were designed so that the same participants attended all three, not all participants from Focus Group Part 1 were able to attend subsequent groups. In this case, we invited new participants who expressed a desire to participate. A total of 20 participants attended the focus groups (male=11; female=9). All participants were of white British ethnicity. Five participants attended all three focus groups; eight attended two focus groups; and seven attended one group. In the first Focus Group Part 1, ten participants attended (male=5; female=5). In the second Focus Group Part 1, five participants attended (male=3; female =2). In Focus Group Part 2, nine participants attended (male=4; female=5). In the first Focus Group Part 3, eight participants attended (male=5; female=3). And, in the second Focus Group Part 3, three participants attended (male=1; female=2).

All participants were asked to provide informed consent before taking part and were able to withdraw at any point. Participants were also assured that their contributions would be pseudonymised during analysis and published outputs would be anonymised. As part of the informed consent process, participants were asked whether they were happy for discussions to be recorded and for recordings to be stored with ALSPAC for potential future use. Participants were not obliged to participate in all three parts of the focus groups and were reimbursed for each participation with a £10 voucher, as well as for any travel expenses incurred. Participants were rewarded with an extra £5 if they participated in all three focus groups. Focus groups took place in the ALSPAC 'focus clinic', which is a study assessment centre.

### Data analysis

Recordings were transcribed by a university-authorised transcription service. Inductive thematic analysis was initially carried out by author KS, which allowed themes to be linked with the data itself, rather than with a pre-existing coding framework ^31^. After becoming familiar with the data, initial codes relating to participants’ concerns and associated safeguards were generated and merged where appropriate. Codes generated included: ‘what will you do with my data’, ‘what data are you collecting’, ‘who will see my data’, ‘what could my data reveal’. Codes were then grouped into themes. All authors reviewed the themes, discussed to what extent they related to the research questions, then developed the final overarching themes: understanding, trust, and control. Data analysis was carried out using NVivo software version 11.4.3.

Wherever possible, original quotes from participants have been included in the analysis in order to minimise any misinterpretations of the data. [FG1] refers to quotes from the first wave of focus groups, [FG2] refers to quotes from the second wave of focus groups, and [FG3] refers to quotes from the third wave.

## Results

This study is a first step towards understanding participants’ attitudes to linking transactional records into longitudinal population studies for public health research. We aimed to investigate (i) whether participants understand why linking loyalty cards and banking data into the study databank is useful for public health research, and if they do, what are the best and most efficient ways of explaining the utility of public health research with transactional data. We further explored (ii) different reactions that respondents might have towards transactional records linkages and the spectrum of individual reactions. Finally, we were interested whether participants are prepared to agree to such data linkage and if so, (iii) given participants’ concerns, what safeguards need to be put in place to make transactional data linkage possible.

### Understanding

We first discuss whether participants understood why there is a need to link their personal data into a longitudinal population study databank, and the most common concerns associated with data linkage. In general, there was a range of opinions on whether sharing personal transactional data was acceptable. Some participants were indifferent about sharing certain types of transactions:

*“I really don’t mind any of the stuff that’s like clothing store, petrol station, food.”* [FG2]

Others raised various concerns about data linkage and data sharing, which we discuss below.

The acceptability of the level of data sharing often appeared to be contingent on the context and purpose of data sharing, which showed that it is important for participants to be informed about the details of data being collected and who the data is shared with. Even though sharing data for commercial purposes was never an option in our scenarios, given the general public narrative around personal data and that this is not permitted within ALSPACs participant framework ^1^, sharing data for private benefit was still discussed at the focus groups. In particular, there appeared to be a misunderstanding with regards to the potential of sharing transactional data with commercial companies or for profit. For example, a common concern was whether credit history, loans or insurance may be affected if companies could access transactional data:

*“Could there be a chance that that might impact the deals you get from your bank maybe?”* [FG1]
*“Will it affect things like your credit history and stuff?”* [FG1]

Individuals were more prepared to share data if they felt it would be beneficial for society, rather than if it is for political or commercial gain, and the general consensus was that donating data to researchers is purposeful:

*“We’ve been giving them [ALSPAC] data since before we were born [...] It’s obviously helped with so many things already.”* [FG2]

For one participant in particular, personal experiences with illness seemed to influence their opinion on the value of transactional data for health research:

*“I had erm, [HEALTH CONDITION, INFORMATION SUPPRESSED FOR DISCLOSURE CONTROL PURPOSES] when I was little and it is literally from the random genetic mutation that no one knows the cause of [...] I think if we can figure things like th*at *out and the data can be useful to do something about that then it has to be worth it.”* [FG2]

However, it was common for participants to be unaware as to how transactional data could be used for research:

*“What are you guys hoping to achieve by understanding what we’re buying, and how is that going to help future generations?”* [FG1]

This suggests that more work needs to be done in explaining the utility of transactional datasets for public health research in an accessible, efficient way: for example, in a similar manner to the Understanding Patient Data initiative led by the National Data Guardian ^2^. One individual highlighted the need for an information sheet explaining the research:

*“I personally would probably give you all of that [transactional data] if I had a sheet explaining that you were going to do something with it and I was happy with the purpose.”* [FG1]

Specifically, there were questions about the potential accuracy of information that can be derived from transactional data linkage, and whether what might be inferred about individuals from their transactional data could be incorrect. This needs to be addressed in any consenting procedures that researchers use for data linkage:

*“But if you’ve got a family you’re going to shop for like all four of you and might not actually drink any of it. So actually it’s not that accurate.”* [FG1]

### Trust

An important factor in the decision whether to share transactional data was who the data is shared with. There was a distinct difference in attitudes to sharing personal data with ALSPAC, whom participants trusted, vs external researchers.****** Several participants alluded to the high levels of trust they placed in the ALSPAC (aka Children of the 90’s) researchers, having been part of the cohort since birth and having experienced high standards of research practice, and they felt happy to provide them with their transactional data:

*“That’s one of the things I really like about Children of the 90’s, they collect all of this data but they keep it anonymous and confidential, always.”* [FG1]

This was also linked in part to the knowledge of how their identity was protected through pseudonymisation processes - referred to as being registered as an ID number:

*“You’re just a number and you know your shopping habits or your banking habits are just part of a bigger data search. That feels, that feels safer doesn’t it?”* [FG2]

One participant also highlighted that they were more trusting of the motivations of ALSPAC researchers:

*“The motivations of Children of the 90’s, some policy makers and people who are researching or looking into rare diseases that would be, erm, I don’t know, it’s something about their motivations just seems more legitimate.”* [FG2]

However, there were general concerns around whether external researchers could be trusted not to misuse data. This fear may have been stoked by an increasing awareness amongst the general public about the power and risks of mis-use of personal data, with participants alluding to Cambridge Analytica scandal as an example of how personal data could be harnessed for political manipulation. The focus groups were conducted only a couple months after this high profile case of social media data mis-use was revealed:

*“The Cambridge Analytica thing that happened recently no one would have put two and two together with that.”* [FG1]
*“Data protection is not how it used to be.”* [FG2]

Furthermore, one participant described how, even if the organisation was known to be trustworthy, it only takes one individual to manipulate data for their research:

*“[...] the controversy especially when people can kind of skewer results as well. They can choose a certain set of people to kind of choose the idea.”* [FG2]

One participants was also worried about losing control over datasets when they are linked; about the security of their data, and who could access it:

*“Where one data, one set of data could then be linked to something else and then be passed onto someone else [...] that’s when you lose track of where your data is going.”* [FG2]

In order to trust third-party data users, participants stated that they would like to be able to access information on who they are sharing information with and how trustworthy the organisation is:

*“Can we trust them? I guess you would look into it wouldn’t you and see what other people have said about them.”* [FG2]
*“I’ve said people are sceptical or have no trust when it comes to data but not around more trustworthy organisations or organisations you have a trust in history.”* [FG2]

Furthermore, precautions should be taken and reassurance should be given to participants that any external researchers approaching longitudinal cohort study participants for data should fulfil the same high standards of research practice as the study itself:

*“So long as they act in the same way that Children of the 90’s do then I don’t see any problem of having the same level of data that you do.”* [FG3]

Fears appeared to be founded on previous experiences with commercial companies not linked with Children of the 90’s or academic research, and more specifically, a lack of consent procedures:

*“I think a lot of research that Children of the 90’s do which other companies don’t do - firstly they don’t give you like a finer consent which Children of the 90’s do. You send out each and every subject you do, every single test you do.”* [FG2]

A participant also highlighted how they would like to be able to verify with Children of the 90’s that they can trust external researchers:

*“I’d probably ring Children of the 90’s and be like ‘what do you think?’”* [FG2]

### Control

Under this theme, we further discuss what are the safeguards and consent procedures that participants suggested to put in place to remedy and address concerns they raised about understanding the purpose of sharing, guarding sensitive types of data and who the data is shared with. Firstly, it is important for participants to have an opportunity to choose what kind of research they donate their data to:

“ *So you always know what your data’s going to be used for and how it’s going to be used and then you can have that choice, that power of stuff to say ‘okay don’t use it for that or yeah okay you can use it.’”* [FG2]

One participant suggested that they could be presented with a range of categories of research that they could opt in or out of:

“ *I think if you opted in and it was under the umbrella of physical and mental health research and innovation.*” [FG2]

Participants highlighted that it is important to explain what ‘transactional data’ means before asking for consent to share the data as individuals felt that being asked to consent in general would cause potential participants to decline involvement in the study as they would prefer to have control over which types of data are shared:

*“If you just say transactional data, if someone doesn’t really want online stuff within that they will just say no to the whole thing. Whereas they might have been happy for the loyalty cards stuff.”* [FG2].

However, they were aware that extracting the data themselves and passing this onto researchers every time could be potentially time-consuming and prevent them from sharing data. Participants suggested one solution for how consent for sharing various types of data could be obtained efficiently, with various categories of data and an accompanying explanation, which they could select if they were happy to share. For instance, there were a number of sensitive categories of data that participants said they were unhappy about sharing, due to fears about what data could reveal about them, such as information geospatial information that can be derived from shopping data:

*“I don’t really think I want people to know where I shop and how often just because, I don’t know, it’s a bit personal.”* [FG3]

Participants also indicated that they considered some types of information to be more private and which they would be reluctant to share. For instance, in Focus Group 1a and 3, two participants indicated that data revealing salary was more private; two participants in focus group 3 believed that some members of the public would be worried whether their data could reveal information about gambling; and one participant in focus group 3 was concerned about sharing transactions related to purchased medicines:

*“I think there will be some people that would have a problem with it. Maybe for things like you don’t want people to know how much you earn or if you’re gambling a lot. So people that have some insecurities maybe don’t want other people to know about it.”* [FG3]
*“I don’t really mind if people know I’m buying shoes or meat and groceries and stuff. But I think the only sensitive, I don’t know whether it should be sensitive or a privacy issue, is prescription medicine, contraception maybe.” [FG3]*

Individuals feared that the more granular the data is, the less likely data would remain anonymous:

*“It depends on how it’s collected and how confidential the data’s going to be, how anonymous the data’s going to be, because like, if you’re looking at us as a wide group and you’re seeing like, one of us bought flowers on a particular date, that’s not really an issue but like, if you’re looking at each individual and you’re seeing personal transactions, that’s more confidential.”* [FG1]

Participants were concerned about information related to other people associated with their transactions that could be revealed through sharing transactional records who had not consented to have their data used in the research:

“ *I think any outgoing payments to specific people, not companies, would be a category because I think most people would probably opt out of that one.”* [FG3]

Transactional data that could reveal details related to children or other minors was agreed to be particularly sensitive:

*“Especially if it’s to do with kids because I wouldn’t want people knowing exactly what school my kids went to or anything like that. I think that one should be completely protected.”* [FG3]

For ALSPAC participants, sharing banking records seemed to make them more nervous:

*“Bank transactions and, I don't really know, stuff like that, if people had that [...] that would stress me out.”* [FG1]

An interesting and unexpected distinction was made between purchases online and those made in stores:

*“If you’re buying something online you might be buying it online because it’s easier or you don’t want people to know what you’re buying.”* [FG2]

Furthermore, the way in which transactional data could potentially reveal business-related information about those who are self-employed was raised by one participant:

*“It depends on lots, like if you’re a business owner or you’re like self-employed or something then you might be a little more, you know, want more of your data kept personal because it affects your business, or like your persona.”* [FG2]

Following discussions on consent for categories, the consensus was that researchers should then extract these types of data according to participants’ wishes:

*“I feel like I personally would rather like have a big list of, I don’t know, ‘can we access this?’ loyalty card, tick yes. And then you do it.”* [FG3]

During the focus groups, we gathered participants’ views on whether requesting permission to access retrospective banking or loyalty card data at any point in time is more acceptable versus requesting permission to access prospective data about an individual’s transactions. Participants raised concerns associated with both future and retrospective data collection, however consenting to share retrospective data collection was seemingly preferable amongst most individuals:

*“I think there’s something in the safety of the past even if it was yesterday.”* [FG3]
*“Because if you say, ‘yeah, you can collect all of my data’ and then, I don’t know, suddenly you lose your job, you start drinking a lot, you don’t anticipate that happening and you might not want people to know. But if you know what’s happened already, you can say ‘yeah, I’m fine for you to collect the last year.’”* [FG3]

However, one participant raised potential issues with retrospective data being collected, remarking:

*“If you were under mental health and in the future say like a year or two years’ time you’re alright, you were working, you’ve got like a family, something like that, you wouldn’t really want that information going out because it was a time of your life which you had done and it could really affect jobs.”* [FG3]

The main issue with providing consent for future data collection appeared to be the way in which researchers could potentially use ‘live’ data to track participants:

*“The other problem with it, with getting future data, is that you could track someone in real time. So you could say that on, there’s a pattern every Tuesday you’re in [LOCATION DETAIL SUPPRESSED FOR DISCLOSURE CONTROL PURPOSES] so that on the next Tuesday someone can be there.”* [FG3]

In general, the notion of ‘live’ data sharing was not comfortable for participants and they wanted information about the concept of ‘live’ data collection and reassurance that they cannot be tracked:

*“People will need that breaking down and explained more I think [...] So, like what does live data mean. Like, how would you go about doing that?”* [FG3]

As regards to forms of consent, participants were largely happy with an initial opt-in consent to extract the records and include them into the ALSPAC resource, but with an ongoing option to opt-out for the subsequent reuse of data:

*“I think we were leaning towards number 3 ^3^. Sort of the information about what it’s going to be used for now and then if you have a big problem with it you can opt out but if you don’t opt out, we’re going to do it anyway.”* [FG2]

Most participants agreed that a yearly check of consent was most appropriate. However, researchers should also make it clear that participants can opt out at anytime, and provide participants with the means to contact them or, as suggested by one participant, by clicking a link online or through an app:

*“Yeah, I’ll probably say yes to anything and everything [...] just as long as I knew what it was and if I did ever want to change my mind, I knew that I was free to do so.”* [FG2]
*“So, I think if we had something we could, like a link or something, we could click on at any point and opt out I think that would be good.”* [FG3]

There was, however, a feeling that this was a futile process linked with a sense of resignation and acceptance from some that data always leaks out and that no one can do anything about this, as this is part of life today. Thus, participants may require reassurance and details relating to data security measures:

*“I don’t think researching who’s taking your data really matters anymore because you have hacks in data and breaches these days.”* [FG2]

Specific reassurances that participants would like included consent, encrypted and anonymous data, and that any identifying information will remain with the ALSPAC team:

*“I don’t think I mind if it it’s anonymous. [...] So long as there’s no way of linking it back to you. [...] If it’s just a number.”* [FG1]

## Discussion

The results of these focus groups represent contributions to the development of ‘ethical parameters’, a process which Metcalf and Crawford ^32^ suggest is crucial as data science methodologies develop. Below we discuss the main findings and associated recommendations.

### Finding 1: A lack of awareness as to why transactional data is valuable for health research

Perhaps unsurprising given the novelty of the topic, there was little to no awareness amongst participants of the value of transactional data for data linkage with their ALSPAC records. This is comparable to findings from a large-scale qualitative study, which found that the public have low levels of understanding about the uses of their patient data for health research ^33^. Attitudes amongst the focus group participants evolved as the proposed usage and benefits that transactional data could bring to ALSPAC research became clearer. Initially, many expressed surprise at the concept and an unfamiliarity as to how this could inform research. Those participants who were cautious about data sharing initially transitioned to a willingness to donate this type of data: a change linked with a desire to help find cures for diseases, or benefit society in more general terms coupled with the clarification of the data processing activities and the safeguards that could be deployed. These findings reflect those of Skatova and Goulding ^16^, who found that willingness to engage in data donation was linked with an understanding of the purpose of the research and the prosocial motive of an individual.

### Recommendation 1

Prior to approaching participants for consent, researchers should consider the need to emphasise the value of transactional data and the potential impact of the proposed research. It is unlikely however, from this evidence based on a small and typically committed participant group, that all ALSPAC participants would accept this use of their data. To reassure those individuals, fair processing information materials would need to also emphasise why this new activity was in keeping with the broader and ‘traditional’ remit of the study; and then to make clear this was optional activity based on explicit consent for those accepting of this use of their data (see Finding 2 below). This otherwise raises concerns that the activity could be perceived as a shift of study direction, which may threaten wider participation and trust.

In turn, this may reinforce the value in approaches where studies (such as ALSPAC) emphasise that ‘enrolment’ does not commit any participant to undertaking any assessment or providing data (i.e. taking part in any assessment is optional, providing an item of data within any assessment is optional). This may encourage a feeling of choice and an acceptance of innovation within study activities even where the activity is not personally acceptable; although this may need to be offset against a potential feeling of obligation to take part by very committed participants. These considerations remain under-explored, but reinforce the need for clear messaging on the purpose of data collection and that taking part is optional. This also highlights the benefits of studies operating parallel ‘innovation’ studies (e.g. the Understanding Society Innovation Panel ^34^) where innovative approaches can be tested in an accepting sample.

However, subsequent discussions showed that although increased knowledge of the uses of transactional data for linkage seemed to encourage positive reactions towards donation, this was also accompanied by a range of concerns and queries about what the process would entail and its potential repercussions, which are described below.

### Finding 2: Participants need to maintain control over personal data sharing for research

A common theme running throughout the focus groups and linked to a number of concerns was the need to maintain control. Bradwell and Gallagher ^35^ point out how individuals ‘surrender control’ when sharing personal information. They suggest that, in order to allow participants to regain control, there should be a move towards a more ‘democratic use of personal information’ with a ‘bottom-up policy driven by collectively negotiated norms and rules’. This reflects previous findings in ALSPAC where participants suggested safeguards relating to the study use of routine health and government records ^4^.

### Recommendation 2

Participants in our study suggested a number of ways in which they could maintain control over their data sharing, linked with various consent mechanisms. Firstly, a number of participants expressed the need for control over the type of research their data is used for, and secondly, control over the types of transactional data they donate, in particular, purchases or transactions they viewed as sensitive, including those involving third-parties. Therefore, researchers should consider providing an opt-in consent list of various categories of research and categories of transactional data.

### Finding 3: There are differences in attitudes to sharing different types of transactional data

When discussing the types of transactions that would be visible to researchers, participants appeared to be split over whether sharing certain types of data would cause them concern and highlighted which parts of their data should not be shared. In particular, participants put more emphasis on protecting their banking than loyalty cards data. The importance of how sensitive the information that can be revealed through shared data influenced different attitudes between sharing loyalty card data and banking records (e.g., salary information from banking records was discussed as very sensitive). Our findings are in line with previous research ^16^ suggesting that people are more concerned about protecting their banking records as compared to loyalty card data when sharing data in general. This concern was sometimes linked with fears that their identity could be exposed, or that behaviours could impact their credit scores.

### Recommendation 3

This finding highlights the need for longitudinal population studies to explain detailed reason for linking different types of transaction data. Further, the difference between different data types is especially important when explaining the process of linking the data, and the ways in which participant identifiers are only visible to the data linkage organisation, in this case ALSPAC, and not external researchers requesting the data ^27^.

### Finding 4: Granularity of the data can affect decisions to share

There was greater concern about data linkage amongst participants when the data appeared more granular. This finding reflects previous research on data sharing ^36^ suggesting that individuals are more likely to assign higher value to protect more granular, less anonymous data whilst making a decision to share personal data with third parties. Furthermore, the same study demonstrated that the general public perceives as less risky sharing personal data with universities for academic research compared to governments for planning or administrative purposes, or private companies for either research or profit-making purposes.

### Recommendation 4

Despite ALSPAC’s wider assurances that participant data will not be used for profit, conversations about sharing personal data do commonly raise fears by association with sharing commercial data for profit and it is important to clarify to participants that this will not take place. This also places emphasis on the need for rigorous data processing pipelines (and the clear description of these) where the transformation of granular and disclosive data to structured data with low disclosure potential is handled by study data managers operating in a trusted role.

### Finding 5: There are differences in attitudes to share retrospective vs prospective data

The concept of ‘live’ data and its association with tracking particularly worried participants, rather than continuous-in-time data sharing, which has little resemblance to ‘live’ tracking. Studies will need to consider if they seek retrospective and/or prospective data collection and explain that “live” data tracking is not required for research purposes. Although the predominant view amongst participants in this study was that they would prefer to donate their data retrospectively rather than prospectively, concerns were expressed towards both.

### Recommendation 5

We suggest that consent forms should provide participants with the opportunity to choose whether to donate retrospective or future transactional data too, with information on the risks associated with both options explained. Similarly, researchers should consider providing options to consent on an opt-in basis and opt-out. Researchers should consider that the most practicable route for extracting these records will be via participants initiating ‘right to portability’ requests which will, by necessity, be opt-in and retrospective at the first instance, but discuss with participants whether they consent to researchers to access future data.

### Finding 6: High levels of trust in a research organization are crucial to encourage data sharing for research

A final common theme running through the focus groups was that of mistrust in the general contemporary use of personal information and digital footprint data. This echoes findings from a recent qualitative study interviewing 2,259 adults online ^37^, where participants portrayed a picture of a society distrusting of data sharing, associated with the increasing awareness of misuse, such as data harvesting. However, participants in our study expressed high levels of trust in ALSPAC staff and were reassured to learn that ALSPAC would carry out the processing and linkage of the data;

### Recommendation 6

Despite the levels of trust participants have in ASLPAC, they will require reassurance that any external researchers using their data will uphold the same standards, particularly in regard to encryption and anonymity.

### Strengths and limitations

According to the authors’ knowledge, this study is the first of its kind to qualitatively explore attitudes towards transactional record sharing and linkage in the context of longitudinal research. The findings therefore are novel and provide an initial step towards the development of an evidence-based conceptual framework guiding researchers looking to recruit participants into transactional record linkage studies. The design of the study involving three focus groups for the same participants allowed time for reflection and to form opinions on this novel field.

Limitations of the study include the small sample size, that all participants were approximately the same age (reflecting the ALSPAC index sample) and the lack of consistent participation of the same individuals across the three focus groups, which meant that more in-depth discussion around the topic may have been limited. The nature of focus groups may have also meant that the expression of participants’ true attitudes was limited by social desirability bias. As participants have been part of the ALSPAC cohort since birth, they were likely to be more informed of the benefits and consequences of sharing their data for research, as well as certain processes, such as de-identification, than the general population, and thus likely to suggest known safeguards. The reported attitudes are likely to be shaped by their continuing trust and acceptance of involvement in longitudinal research; this was not a representative group of all study participants, and the value of these insights lies more in terms of helping inform process design and communication strategies rather than as an indication of how many participants would accept this use of their data. Therefore, the results are mostly relevant to longitudinal population studies participants rather than the wider general public. Finally, it is plausible that because we discussed different types of data with participants, and specifically different types of transactional data, the results are biased by the order in which different data types were discussed. For example, participants might be more likely to express positive attitudes about data linkage in general if and when loyalty cards data were presented to them before banking data, and vice versa. Due to sample size we do not have sufficient evidence to provide conclusive evidence on this matter.

### Future research

Future research should seek to explore the views of a more diverse range of participants from the general population towards the donation of transactional records for public health research to provide a more generalisable picture of attitudes. Follow-up interviews could also complement focus group discussions by providing an opportunity for researchers to discuss any specific issues arising from focus groups in more depth and explore any variations in opinions following a period of reflection. The use of individual qualitative interviews could enable participants to express their insights in more detail, whilst reducing the possibility of bias introduced by group consultations. The contrasts between participants’ views on loyalty card data and banking data could also be a topic for future research. In particular, it would be of interest to investigate whether opinions on the use of one type transactional of data (i.e. loyalty card data) could affect an individual’s views on the other type of transactional data (i.e. banking data). This could also be explored in a quantitative survey, where the type of data participants are asked about first is randomised.

## Conclusions

This study provides initial evidence on the attitudes and concerns of participants currently involved in a longitudinal cohort study towards providing their loyalty card and banking transactional records into the study databank. The findings suggest a number of safeguards which researchers should consider when looking to recruit participants for similar studies. Across the three waves of workshops, participants went on a ‘journey’ of first seeking to understand the purpose behind the linkage of their transactional records with their previously collected ALSPAC data, and the purpose of ALSPAC research; then discussing their concerns; and finally, suggesting safeguards needed to make this form of data linkage acceptable. In particular, researchers seeking to recruit participants into transactional data linkage studies should consider the importance of ensuring participants have access to appropriate information on data usage, control over their data, and trust in the organisation.

## Data availability

### Underlying data

ALSPAC data access is through a system of managed open access. The steps below highlight how to apply for access to the data included in this research article and all other ALSPAC data. The datasets presented in this article are linked to ALSPAC project number B3021, please quote this project number during your application. The ALSPAC variable codes highlighted in the dataset descriptions can be used to specify required variables.

1. Please read the ALSPAC access policy ( https://www.bristol.ac.uk/media-library/sites/alspac/documents/researchers/data-access/ALSPAC_Access_Policy.pdf) which describes the process of accessing the data and samples in detail, and outlines the costs associated with doing so.
2. You may also find it useful to browse our fully searchable research proposals database ( https://proposals.epi.bristol.ac.uk/), which lists all research projects that have been approved since April 2011.
3. Please submit your research proposal for consideration by the ALSPAC Executive Committee. You will receive a response within 10 working days to advise you whether your proposal has been approved.

If you have any questions about accessing data, please email alspac-data@bristol.ac.uk.

The ALSPAC data management plan describes in detail the policy regarding data sharing, which is through a system of managed open access.

The study website also contains details of all the data that is available through a fully searchable data dictionary: http://www.bristol.ac.uk/alspac/researchers/data-access/data-dictionary/.

## Note


^1^ https://www.bristol.ac.uk/alspac/participants/our-commitment-to-you/

^2^ https://www.understandingpatientdata.org.uk

^3^Personal transactional data can be linked with data previously collected by Children of the 90s and used for academic research with opt-in consent. These records can be re-used for different C90s projects, and participants have the right to object (opt-out) and stop this from happening.

## References

[1] Shlomo N : Overview of Data Linkage Methods for Policy Design and Evaluation.In: Crato N, Paruolo P (eds.). *Data-Driven Policy Impact Evaluation.* Springer, Cham.2018;47–65. 10.1007/978-3-319-78461-8_4

[2] da Silva ME Coeli CM Ventura M : Informed consent for record linkage: a systematic review. *J Med Ethics.* 2012;38(10):639–642. 10.1136/medethics-2011-100208 22403083

[3] Lynn P : Methods for Longitudinal Surveys.In: Lynn P (ed.). *Methodology of Longitudinal Surveys.* West Sussex, John Wiley & Sons Ltd.2009;1–18. 10.1002/9780470743874.ch1

[4] Audrey S Brown L Campbell R : Young people's views about consenting to data linkage: findings from the PEARL qualitative study. *BMC Med Res Methodol.* 2016;16:34. 10.1186/s12874-016-0132-4 27001504 PMC4800768

[5] Sedgwick P : Bias in observational study designs: prospective cohort studies. *BMJ.* 2014;19(349):g7731. 10.1136/bmj.g7731 25527114

[6] Davis-Kean P Chambers RL Davidson LL : 2017 Report to the Economic and Social Research Council.ESRC Longitudinal Studies Strategic Review.2017; Accessed 23 October 2019. Reference Source

[7] Medical Research Council: Maximising the value of UK population cohorts. MRC Strategic Review of the Largest UK Population Cohort Studies.2014; Accessed 23 October 2019. Reference Source

[8] Wellcome’s Longitudinal Population Studies Working Group: Longitudinal Population Studies Strategy.2017; Accessed 23 October 2019. Reference Source

[9] Boyd A Gatewood J Thorson S : Data Diplomacy. *Science & diplomacy.* 2019;8(1). Reference Source PMC6785044 31598426

[10] Health Data Research UK: One Institute Strategy 2019/20.2019; Accessed 23 October 2019. Reference Source

[11] McGrail K Jones K Akbari A : A position statement on population data science. *Int J Popul Data Sci.* 2018;3(1). 10.23889/ijpds.v3i1.415 PMC8142960 34095517

[12] Regulation (EU) 2016/679 of the European Parliament and of the Council of 27 April 2016 on the protection of natural persons with regard to the processing of personal data and on the free movement of such data, and repealing Directive 95/46/EC (General Data Protection Regulation).Accessed 16 September 2019. Reference Source

[13] van Ooijen I Vrabec HU : Does the GDPR Enhance Consumers’ Control over Personal Data? An Analysis from a Behavioural Perspective. *J Consumer Policy.* 2019;42(1):91–107. 10.1007/s10603-018-9399-7

[14] Weaver S Gahegan M : Constructing, visualizing and analyzing a digital footprint. *Geogr Rev.* 2010;97(3):324–350. 10.1111/j.1931-0846.2007.tb00509.x

[15] Quinn P : Is the GDPR and Its Right to Data Portability a Major Enabler of Citizen Science? *Glob Jurist.* 2018;18(2). 10.1515/gj-2018-0021

[16] Skatova A Goulding J : Psychology of personal data donation. *PLoS One.* (in press). Reference Source 10.1371/journal.pone.0224240 PMC6867598 31747408

[17] Aitken M de St Jorre J Pagliari C : Public responses to the sharing and linkage of health data for research purposes: a systematic review and thematic synthesis of qualitative studies. *BMC Med Ethics.* 2016;17(1):73. 10.1186/s12910-016-0153-x 27832780 PMC5103425

[18] Stockdale J Cassell J Ford E : "Giving something back”: A systematic review and ethical enquiry into public views on the use of patient data for research in the United Kingdom and the Republic of Ireland [version 2; peer review: 2 approved]. *Wellcome Open Res.* 2019;3:6. 10.12688/wellcomeopenres.13531.2 30854470 PMC6402072

[19] Jones K Daniels H Heys S : Public Views on Using Mobile Phone Call Detail Records in Health Research: Qualitative Study. *JMIR Mhealth Uhealth.* 2019;7(1):e11730. 10.2196/11730 30664467 PMC6352010

[20] Damschroder LJ Pritts JL Neblo MA : Patients, privacy and trust: patients' willingness to allow researchers to access their medical records. *Soc Sci Med.* 2007;64(1):223–235. 10.1016/j.socscimed.2006.08.045 17045717

[21] Davidson S McLean C Ipsos MORI Scotland, : Public Acceptability of Cross-Sectoral Data Linkage: Deliberative research findings. Scottish Government Social Research, Edinburgh.2012; Accessed 09 October 2019. Reference Source

[22] Medical Research Council, Ipsos MORI: The Use of Personal Health Information in Medical Research. General Public Consultation. Final Report.2007; Accessed 23 October 2019. Reference Source

[23] Weitzman ER Kaci L Mandl KD : Sharing medical data for health research: the early personal health record experience. *J Med Internet Res.* 2010;12(2):e14. 10.2196/jmir.1356 20501431 PMC2956225

[24] Wellcome Trust: Enabling data linkage to maximise the value of public health research data.Final report to The Wellcome Trust.2015; Accessed 23 October 2019. Reference Source

[25] Ioannidis JPA : Informed consent, big data, and the oxymoron of research that is not research. *Am J Bioeth.* 2013;13(4):40–42. 10.1080/15265161.2013.768864 23514395

[26] Audrey S Brown L Campbell R : Young people's views about consenting to data linkage: findings from the PEARL qualitative study. *BMC Med Res Methodol.* 2016;16:34. 10.1186/s12874-016-0132-4 27001504 PMC4800768

[27] Xafis V : The acceptability of conducting data linkage research without obtaining consent: lay people’s views and justifications. *BMC Med Ethics.* 2015;16(1):79. 10.1186/s12910-015-0070-4 26577591 PMC4647472

[28] Boyd A Golding J Macleod J : Cohort Profile: the 'children of the 90s'--the index offspring of the Avon Longitudinal Study of Parents and Children. *Int J Epidemiol.* 2013;42(1):111–127. 10.1093/ije/dys064 22507743 PMC3600618

[29] Fraser A Macdonald-Wallis C Tilling K : Cohort Profile: The Avon Longitudinal Study of Parents and Children: ALSPAC mothers cohort. *Int J Epidemiol.* 2013;42(1):97–110. 10.1093/ije/dys066 22507742 PMC3600619

[30] Northstone K Lewcock M Groom A : The Avon Longitudinal Study of Parents and Children (ALSPAC): an update on the enrolled sample of index children in 2019 [version 1; peer review: 2 approved]. *Wellcome Open Res.* 2019;4:51. 10.12688/wellcomeopenres.15132.1 31020050 PMC6464058

[31] Braun V Clarke V : Using thematic analysis in psychology. *Qual Res Psychol.* 2008;3(2):77–101. 10.1191/1478088706qp063oa

[32] Metcalf J Crawford K : Where are human subjects in Big Data research? The emerging ethics divide. *Big Data Soc.* 2016;3(1). 10.1177/2053951716650211

[33] Ipsos MORI, Wellcome Trust: The one-way mirror: public attitudes to commercial access to health data.2016; Accessed on 03 October 2019. Reference Source

[34] Uhrig SN : Using experiments to guide decision making in Understanding Society: Introducing the Innovation Panel. *Understanding Society.* 2011. Reference Source

[35] Bradwell Gallagher : FYI: The new politics of personal information.2007; Accessed 03 Oct 2019. Reference Source

[36] Mcdonald R Skatova A Ma S : Willingness to Share Personal Data Online - a Discrete Choice Experiment. (in preparation).

[37] Information Commissioner’s Office: Information Rights Strategic Plan: Trust and Confidence.2019; Accessed 23 October 2019. Reference Source

