# Supplementary material for: Attitudes towards transactional data donation and linkage in a longitudinal population study: evidence from the Avon Longitudinal Study of Parents and Children
Source: Wellcome Open Res. 2021 Jun 9;4:192. Originally published 2019 Dec 3. [Version 2] doi: 10.12688/wellcomeopenres.15557.2 (PMC7341003; doi:10.12688/wellcomeopenres.15557.2)
Supplement: Peer review history

# Peer review: Reviewer response for version 2

**DOI:** 10.21956/wellcomeopenres.18120.r44324

**Published:** 2021-06-10

**Authors:** Vicki Xafis

**Affiliations:** 1 Centre for Biomedical Ethics, Yong Loo Lin School of Medicine, National University of Singapore, Singapore, Singapore; 2 School of Public Health, Sydney Health Ethics, The University of Sydney, Sydney, Australia; 3 Faculty of Health and Medical Sciences, The University of Adelaide, Adelaide, Australia; 4 School of Women's and Children's Health, The University of New South Wales, Sydney, Australia

Thank you for the revisions. Changes to the Discussion are very helpful.

Is the work clearly and accurately presented and does it cite the current literature?

Yes

If applicable, is the statistical analysis and its interpretation appropriate?

Not applicable

Are all the source data underlying the results available to ensure full reproducibility?

Yes

Is the study design appropriate and is the work technically sound?

Yes

Are the conclusions drawn adequately supported by the results?

Yes

Are sufficient details of methods and analysis provided to allow replication by others?

Yes

Reviewer Expertise:

Bioethics, Qualitative Research, Linguistics

I confirm that I have read this submission and believe that I have an appropriate level of expertise to confirm that it is of an acceptable scientific standard.

# Peer review: Reviewer response for version 1

**DOI:** 10.21956/wellcomeopenres.17038.r39311

**Published:** 2020-07-07

**Authors:** Kerina Jones

**Affiliations:** 1 Population Data Science, Swansea University Medical School, Swansea University, Swansea, UK

This is a very interesting and thorough study addressing attitudes towards transactional data donation and linkage among participants of the ALSPAC study. The work has been conducted comprehensively, from the introduction setting out the background and rationale for the work, to the interpretation of the results and their relevance.

 I have some comments for suggested improvements and clarifications:

 The authors identify various transactional data and choose to focus on retail loyalty cards and banking cards data. The reasons for this need to be justified.

 Also in relation to this focus, it would be useful to note observed contrasts between views on the use of retail cards vs banking cards and to comment on whether by having both considered, and only those two types, the opinions on the use of one type of data may have affected the opinions on the use of the other type.

 Information is given on the ALSPAC cohort, which is good. But information is also needed on the sample of 20 people who took part e.g. male/female proportions, ethnicity and other sample characteristics as available.

 In several places the authors use phrases like ‘a number of’ participants. Is it possible to be more specific to provide more information on how many/what proportion of the group in question were of a particular view?

 While not looking to plug my own work, I have carried out public engagement on the use of mobile phone data and the development of an ethically founded framework for the use of such data for health research. This might be of interest to the authors since they are focusing on different types of transactional data ^1^^,^^2^. 

 The authors might like to break up the Discussion into sub-headings, such as Main findings; What this study adds; and Recommendations.

Is the work clearly and accurately presented and does it cite the current literature?

Yes

If applicable, is the statistical analysis and its interpretation appropriate?

Not applicable

Are all the source data underlying the results available to ensure full reproducibility?

Yes

Is the study design appropriate and is the work technically sound?

Yes

Are the conclusions drawn adequately supported by the results?

Yes

Are sufficient details of methods and analysis provided to allow replication by others?

Yes

Reviewer Expertise:

Data sharing platforms; Information governance and data ethics; Public engagement on the use of various types of data for research.

I confirm that I have read this submission and believe that I have an appropriate level of expertise to confirm that it is of an acceptable scientific standard.

## References

[1] : Public Views on Using Mobile Phone Call Detail Records in Health Research: Qualitative Study. *JMIR mHealth and uHealth* .2019;7(1) : 10.2196/11730 10.2196/11730 PMC635201030664467

[2] : Toward an Ethically Founded Framework for the Use of Mobile Phone Call Detail Records in Health Research. *JMIR mHealth and uHealth* .2019;7(3) : 10.2196/11969 10.2196/11969 PMC645047430900996

# Response

**Published:** 2020-11-19

**Authors:** Anya Skatova

**Affiliations:** University of Bristol, UK

We thank the reviewer for insightful comments. Below is our point by point response. 

 1.The authors identify various transactional data and choose to focus on retail loyalty cards and banking cards data. The reasons for this need to be justified.

 -We focused on loyalty card and banking data as we are planning to request this specific data in the future for data linkage. This has now been explained on page 6.

 2.  Also in relation to this focus, it would be useful to note observed contrasts between views on the use of retail cards vs banking cards and to comment on whether by having both considered, and only those two types, the opinions on the use of one type of data may have affected the opinions on the use of the other type.

 -Under finding 3 on page 17 of the discussion, we compare participant views on sharing loyalty card and banking data. We weren’t directly able to study whether opinions on one type of data may have affected the opinions on the other type of data in this study. However, this would make an interesting topic for a future study using a quantitative survey design, where we could control which type of data participants are asked about sharing first. We discuss this point now in the Limitations section.

 3. Information is given on the ALSPAC cohort, which is good. But information is also needed on the sample of 20 people who took part e.g. male/female proportions, ethnicity and other sample characteristics as available.

 -We have now described the gender and ethnicity of participants who took place on page 8.

 4. In several places the authors use phrases like ‘a number of’ participants. Is it possible to be more specific to provide more information on how many/what proportion of the group in question were of a particular view?

 -We have now quantified ‘a number of’ on page 13 and removed phrases like ‘many’ in other parts of the manuscript.

 5. While not looking to plug my own work, I have carried out public engagement on the use of mobile phone data and the development of an ethically founded framework for the use of such data for health research. This might be of interest to the authors since they are focusing on different types of transactional data ^1^ ^,^ ^2^ . 

 -Thank you for directing us to this research. We have now referenced this in our introduction on page 4.

 6. The authors might like to break up the Discussion into sub-headings, such as Main findings; What this study adds; and Recommendations.

 -We have broken up the discussion into six ‘findings’ and associated ‘recommendations’ in order to make this clearer for the reader.

# Peer review: Reviewer response for version 1

**DOI:** 10.21956/wellcomeopenres.17038.r37264

**Published:** 2019-12-11

**Authors:** Vicki Xafis

**Affiliations:** 1 Centre for Biomedical Ethics, Yong Loo Lin School of Medicine, National University of Singapore, Singapore, Singapore; 2 School of Public Health, Sydney Health Ethics, The University of Sydney, Sydney, Australia; 3 Faculty of Health and Medical Sciences, The University of Adelaide, Adelaide, Australia; 4 School of Women's and Children's Health, The University of New South Wales, Sydney, Australia

Thank you very much for the opportunity to review this paper. This was a very well-conceived research project and the focus group sessions were creatively designed. The paper is also very well written and easy to follow. It was a pleasure reading it.

 The project focuses on an increasingly important but under-explored topic relating to large amounts of data we generate as we go about our daily lives (transactional data) and considers its use in longitudinal cohort studies. The research adds to the body of work around public perceptions about, and preferences for sharing data and the linkage of data from different sources and areas of life. The authors acknowledge that individual awareness of the value of this kind of data is low and the research participants confirmed this with their queries about how such data would be useful in health research. The overarching themes identified relate to participants' *Understanding*, *Trust*, and *Control *over uses of transactional data via standard consent mechanisms. This cohort in effect formed a 'community' as a result of their long-term involvement with ALSPAC and expressed great trust towards these researchers and ALSPAC. This seems to have greatly influenced their willingness to share transactional data with ALSPAC but broader reservations were evident.

 Some suggestions which may improve an already excellent paper include the following:

1. Readers would benefit from more detail about ALSPAC, particularly the fact that external researchers can request access to data held in the ALSPAC data repository for research purposes. This was not immediately clear and required the reader to make assumptions where there were references to ‘external researchers’.
2. It was not clear if written materials were made available to participants, particularly as they were intended to attend all focus group discussions. I have assumed that this was not the case.
3. P6. “…in order to minimise authors’ interpretation of the data” should perhaps be ‘misinterpretation’, as qualitative research does require the researcher to interpret the data.
4. P7. The last section in the first column under ‘Trust’ could be moved to the section titled ‘Control’.
5. Readers would benefit from some detail around the envisaged process of initial donation of transactional data. A brief description of this process would add greater clarity and would add valuable contextual information pertinent to the issues raised and discussed.
6. One thing that is obvious, but not mentioned in the Discussion, is the age of this cohort, which could perhaps also have some bearing on the views expressed. This is something that should perhaps be explored in future research relating to the use of transactional data.
7. Overall, despite the great trust that this cohort quite evidently has in ALSPAC and its researchers, there seem to be more concerns about the sharing of transactional data than a willingness to share such data for beneficial health research. I was wondering if you agree with this and, if so, whether a broad statement of this kind would be useful in the Discussion.

Is the work clearly and accurately presented and does it cite the current literature?

Yes

If applicable, is the statistical analysis and its interpretation appropriate?

Not applicable

Are all the source data underlying the results available to ensure full reproducibility?

Yes

Is the study design appropriate and is the work technically sound?

Yes

Are the conclusions drawn adequately supported by the results?

Yes

Are sufficient details of methods and analysis provided to allow replication by others?

Yes

Reviewer Expertise:

Bioethics, Qualitative Research, Linguistics

I confirm that I have read this submission and believe that I have an appropriate level of expertise to confirm that it is of an acceptable scientific standard.

# Response

**Published:** 2020-11-19

**Authors:** Anya Skatova

**Affiliations:** University of Bristol, UK

We thank the reviewer for insightful comments. Below is our point by point response. 

 1.Readers would benefit from more detail about ALSPAC, particularly the fact that external researchers can request access to data held in the ALSPAC data repository for research purposes. This was not immediately clear and required the reader to make assumptions where there were references to ‘external researchers’.

 - We have now added the following comment on page 6: ALSPAC is a globally accessible research resource, which also allows recall studies: including those considering participant understanding, expectations and acceptability of different research designs. The ALSPAC resource has an online data dictionary (URL) and a public access mechanism ( http://www.bristol.ac.uk/alspac/researchers/access/).

 2.It was not clear if written materials were made available to participants, particularly as they were intended to attend all focus group discussions. I have assumed that this was not the case.

 -Written materials were not provided to participants prior to the focus groups. We have clarified this in the paper, p 7.

 3.P6. “…in order to minimise authors’ interpretation of the data” should perhaps be ‘misinterpretation’, as qualitative research does require the researcher to interpret the data.

 -This has now been changed to ‘in order to minimise authors’ misinterpretations of the data’.

 4.P7. The last section in the first column under ‘Trust’ could be moved to the section titled ‘Control’.

 -We agree that this section has elements that span sections. However, we consider that this quote would be better placed under ‘trust’ as in this theme we discuss participant concerns about who the data is shared with (specifically ALSPAC vs external researchers), and this specific quote refers to one participant’s fears around what could happen when one set of data is linked with another set of data, and who will be able to see this data. Under the theme ‘control’, we focus more on the safeguards suggested by participants.

 5.Readers would benefit from some detail around the envisaged process of initial donation of transactional data. A brief description of this process would add greater clarity and would add valuable contextual information pertinent to the issues raised and discussed.

 -We feel that this is out of scope for this paper and this research: which was designed to identify factors to incorporate into the donation and processing of the data.

 6.One thing that is obvious, but not mentioned in the Discussion, is the age of this cohort, which could perhaps also have some bearing on the views expressed. This is something that should perhaps be explored in future research relating to the use of transactional data.

 - We fully agree. At the end of the strengths and limitations section on page 18-19, we have now raised this issue, and we mention the importance of including a more diverse range of participants in order to ensure generalisability in the future research section.

 7.Overall, despite the great trust that this cohort quite evidently has in ALSPAC and its researchers, there seem to be more concerns about the sharing of transactional data than a willingness to share such data for beneficial health research. I was wondering if you agree with this and, if so, whether a broad statement of this kind would be useful in the Discussion.

 -This is an interesting observation, and while we agree that many concerns and pragmatic issues were raised, the data suggests that participants were positive about sharing transactional data for health research where appropriate controls are deployed. The discussion was naturally focused around concerns, not benefits, as those were focus of the study. Finally, we found that at the beginning of the focus groups, participants were reluctant to share transactional data, which was linked with a limited understanding of how this novel form of data could be useful for health research. However, once they understood how this data could benefit the public good, participants moved to being in favour of sharing it and the rationale for asking: although this would not suggest all would consent to this use of their data. We address this at the start of the discussion.
